# Serum cystatin C, kidney injury molecule-1, neutrophil gelatinase-associated lipocalin, klotho and fibroblast growth factor-23 in the early prediction of acute kidney injury associated with sepsis in a Chinese emergency cohort study

**DOI:** 10.1186/s40001-022-00654-7

**Published:** 2022-03-11

**Authors:** Yuanyuan Pei, Guangping Zhou, Pengfei Wang, Fang’e Shi, Xiaolu Ma, Jihong Zhu

**Affiliations:** Emergency Department, Peking University People’s Hospital, No.11 Xizhimen South Street, Beijing, 100044 China

**Keywords:** Sepsis, Acute kidney injury, Biomarkers, Cystatin C, KIM-1

## Abstract

**Background:**

Acute kidney injury (AKI) is a common and critical complication of sepsis, and is associated with unacceptable morbidity and mortality. Current diagnostic criteria for AKI was insensitive for early detection. Novel biomarkers including cystatin C, kidney injury molecule-1 (KIM-1), neutrophil gelatinase-associated lipocalin (NGAL), klotho and fibroblast growth factor-23 (FGF-23) can predict AKI earlier and allow immediate interventions. We aimed to determine the diagnostic performance of these biomarkers for detecting AKI in sepsis patients.

**Methods:**

This prospective observational study was conducted between May 2018 and November 2020, enrolling 162 sepsis patients eventually. The AKI was defined in accordance with 2012 KDIGO criteria and we divided patients into non-AKI (*n* = 102) and AKI (*n* = 60) groups. Serum levels of several AKI biomarkers were detected by ELISA. The relationship between biomarker levels on admission of AKI was analyzed and discrimination performances comparison were performed.

**Results:**

AKI incidence was up to 37.0% (60/162) during hospitalization. Compared with non-AKI group, both serum cystatin C, KIM-1, NGAL and FGF-23 were significantly elevated at admission in septic AKI patients. The areas under the receiver operating curves demonstrated that serum cystatin C had modest discriminative powers for predicting AKI after sepsis, and cystatin C combined with serum creatinine in the prediction of septic AKI increased the diagnostic sensitivity prominently.

**Conclusion:**

Serum cystatin C, KIM-1, NGAL and FGF-23 levels were both increased in septic AKI patients. Our study provided reliable evidence that cystatin C solely and combined with serum creatinine may accurately and sensitively predict septic AKI of patients on admission.

## Introduction

Sepsis is a well-known life-threatening syndrome caused by a dysregulated host response to infection, which could lead to multiple organ dysfunction and high fatality [1, 2]. Sepsis has become a global public health problem. There are approximately 30 million sepsis patients worldwide each year with numerous and heavy medical costs [1, 3]. Acute kidney injury (AKI) is a common and critical complication of sepsis, with an incidence from 33 to 50% [2, 3]. As well as sepsis, AKI is independently associated with increased morbidity and mortality, length of stay, and cost of care, so early detection is crucial to increasing the possibility of successful intervention [1–4].

The pathophysiological mechanisms of septic AKI remain complicated and elusive. The diagnosis of AKI is presently based on an increase of serum creatinine (SCr) concentration or a decrease in urine output [5]. The initial limitation of a definition that relies on dynamic change in SCr is establishing a baseline SCr. Meanwhile, SCr is not an accurate indicator for the evaluation of renal function, which is a small molecular weight (113 Da) molecule freely filtered by the glomerulus and reabsorbed minimally by the renal tubules [6]. SCr increases proportionally to muscle mass, and hence varies with age, sex, ethnic group, and extreme diets [6]. Urine output is insensitive and in most cases can only be measured accurately in the intensive care unit (ICU). For sepsis patients, oliguria appears to carry elevated significance, even by 3 to 5 h, an association between oliguria and AKI can be detectable [7, 8]. Therefore, it is urgent for better biomarkers of AKI prediction to improve the prognosis of sepsis patients.

Recently, several biomarkers of early structural kidney injury such as cystatin C, kidney injury molecule-1 (KIM-1), neutrophil gelatinase-associated lipocalin (NGAL), klotho and fibroblast growth factor-23 (FGF-23) have been reported that they may identify early AKI before a significant increase in SCr level, but are not widely used in septic AKI [2, 9].

Cystatin C is a cysteine protease inhibitor which has a half-life of 1.5 h compared with SCr’s 4 h half-life [6, 10]. Therefore, after kidney injury, cystatin C concentration could elevate earlier than SCr, so that earlier diagnosis of AKI is detectable [6, 10]. KIM-1 is a type 1 transmembrane glycoprotein expressed at high levels on the renal proximal tubules in the kidney [2, 6]. NGAL is a 25-kDa protein of the lipocalin superfamily, originally isolated from human neutrophils [2], which increases in the plasma and urine 24 ~ 48 h before the elevation of SCr [11, 12]. Klotho-FGF-23 axis is reported both as AKI’s biomarkers and potential therapies lately [9, 13]. Klotho is mainly expressed in the kidney distal tubule and FGF-23 is its upstream regulator [9, 13]. However, it is not only unique to kidney, but also produced by other tissues.

The emergency department/room (ER) is the first consultation department for the vast majority of sepsis patients and many of them receive treatment in ER. Research in the United States show that more than 500,000 patients suspected of sepsis are treated in ER every year [13]. Nevertheless, current studies on sepsis are mainly conducted in the ICU, and data from ER are scarce. As ER is a window department, early diagnosis of septic AKI in it is more consequential and ponderable. Therefore, we performed a prospective study with sepsis patients in order to explore early biomarkers for AKI prediction at hospital admission and these biomarkers included measurements of glomerular function (cystatin C), proximal and distal tubule function (KIM-1 and klotho).

## Methods

### Study population

The prospective observational study was conducted in the Peking University People’s Hospital from China which included all 195 consecutive patients diagnosed with sepsis between May 2018 and November 2020 in emergency department, and enrolled 162 patients eventually. The following patients were excluded: (1) patients under the age of 18 years (3 cases); (2) those with chronic kidney disease 3 to 5 stages (9 cases), defined according to the definition of the National Kidney Foundation as kidney damage or GFR of less than 60 ml/min per 1.73 m^2^ for at least 3 months [3]; (3) Child–Pugh classification [14] of liver function was at B or C stage (4 cases); (4)with end-stage of malignant tumor (4 cases); (5) who died or were discharged within 48 h of admission (13 cases). Our study has been approved by the hospital ethics committee (No. 2018PHB157-01). Patients or their family members were fully informed of the study details and signed the informed consent forms of their own accord.

### Definitions

Sepsis and septic shock were defined according to the Third International Consensus Definitions for Sepsis and Septic Shock (Sepsis.3.0) [1]. The quick Sequential Organ Failure Assessment (qSOFA) of patients was conducted on admission, which used three categories, assigning one point for low blood pressure (SBP ≤ 100 mmHg), high respiratory rate (≥ 22 breaths per min), or altered mentation (Glasgow coma scale < 15) [1]. And according to the 2012 KDIGO criteria, which was based on the RIFLE/AKIN definitions, we used the urine output and SCr components as indicates of AKI [5]. The AKI is characterized by an increase in serum SCr of 0.3 mg/dL within 48 h, an elevation on to 1.5-fold the baseline level within the first 7 days, or a decline in urine output to not more than 0.5 mL/kg per hour for at least 6 h. The stage of AKI was in accordance with 2012 KDIGO criteria uniformly [5].

### Collection of clinical data

The patient records were reviewed to obtain comprehensive data on the baseline characteristics. Age, gender, co-morbidities, initial vital signs, the peak of lactate and PaO2/FiO2 were collected. To determine the severity of inflammation, the peak of white blood cell (WBC) count, neutrophils (NE), C-reactive protein (CRP) and procalcitonin (PCT) were recorded. In addition, we also recorded the patients’ heart, liver and coagulation function and etiological examination laboratory indicators. Antibiotic and antiviral treatment, use of vasoactive drugs, glucocorticoid and non-steroidal anti-inflammatory drugs (NSAIDs) medication and diuretics were recorded. The maximum daily doses of intravenous loop diuretics were all converted to furosemide, expressed as 1 mg bumetanide ≈ 20 mg torsemide ≈ 40 mg furosemide. Meanwhile, use of central vein catheterization (CVC), mechanical ventilation, renal replacement therapy, extracorporeal membrane oxygenation (ECMO) were also included.

### Serum sampling and biomarker analyses

Serum sample for cystatin C, KIM-1, NGAL, klotho and FGF-23 were taken on the first time when patients diagnosed with sepsis at admission in emergency. SCr levels were detected on admission and every 24 h in the first 7 days. All samples were centrifuged at 1500 rpm for 10 min, and then stored at − 80 °C until detection. All biomarkers were measured in duplicate by a single enzyme-linked immunosorbent assay (ELISA) according to the instructions of manufacturer. NGAL ELISA kits were from Abcam (ab113326), cystatin C ELISA Kits from Sigma (RAB0105), KIM-1 ELISA kits from R&D Systems (DSKM100), Klotho ELISA kits from R&D Systems (DY5334-05) and FGF-23 ELISA kits from R&D Systems (DY2604-05). The measured results were compared between patients with and without AKI. Laboratory investigators were all blind to the clinical information throughout the study.

### Statistical analysis

The primary analysis compared the AKI group with the non-AKI group. All variables were tested for a normal distribution through the Kolmogorov–Smirnov test. All descriptive statistics were summarized and displayed as the mean ± standard deviation or the median (25 ~ 75%). Continuous variables and normal distribution data were compared using independent sample t tests. And continuous variables that not normally distributed were compared by using the Mann–Whitney U test. Categorical data were tested by the Chi-square test or Fisher’s exact test. *P* < 0.05 was considered to be statistical significance.

Receiver operating characteristic (ROC) analysis was used to explore the ability of these biomarkers to predict AKI occurrence in patients with sepsis at admission. Discrimination was assessed based on the area under a receiver operating characteristic curve (AUROC). We described AUCs using the following values: 0.90 ~ 1.0 excellent, 0.80 ~ 0.89 good, 0.70 ~ 0.79 fair, 0.60 ~ 0.69 poor and 0.50 ~ 0.59 no useful performance [15]. The analysis of AUROC was also conducted to estimate the cut-off values, sensitivity and specificity, and cut-off points were calculated by determining the best Youden index. All analyses were performed with SPSS 25.0 software.

## Results

### Study population and incidence of AKI

Overall, 195 consecutive patients were screened. Of these, 33 patients were later excluded according to the exclusion criteria and 162 patients enrolled in this study eventually. Among them, 37.0% (60/162) patients developed AKI in the first 7 days according to the 2012 KDIGO definition. Of the 60 AKI patients, 17 patients (28.3%) developed stage 1 AKI, 14 (23.3%) developed stage 2 AKI, 29 (48.3%) developed stage 3 AKI and 6 received renal replacement therapy. Compared to non-AKI group, the in-hospital mortality of the AKI group was obviously higher (42.7% vs 11.8%, *P* < 0.001).

### Baseline characteristics

The demographic baseline clinical characteristics of the patients are listed in Table 1. Compared to the non-AKI group, the AKI group tends to have higher incidence of chronic heart disease (CHD) and chronic kidney disease (CKD). And patients with AKI had higher qSOFA score and Lac at admission. On auxiliary examinations, AKI group had more serious inflammation responses, worse cardiac and renal function, and severer coagulation system disorders, which manifested as significant increases in CRP, WBC, neutrophils (NE) and PCT, higher levels of BNP, BUN and SCr and lower eGFR level, and the decrease of prothrombin activity and obvious elevation of d-dimer levels. However, it was worth pointing out that there is no statistical difference in patients' LVEF. CVC and mechanical ventilation and CRRT were apparently higher in AKI groups due to severer conditions. Meanwhile, more use of carbapenems and antifungal treatment, more use of vasoactive drugs including noradrenaline and metaraminol and larger doses of diuretics were observed in AKI group.

**Table 1:** Baseline characteristics in sepsis patients

Variables	Total (*n* = 162)	AKI group (*n* = 60)	Non-AKI group (*n* = 102)	*P* value
*Demography*				
Age (years)	72 (58.83)	75 (59.84)	70 (58.83)	0.324
Male (%)	97 (59.9)	39 (65.0)	58 (56.9)	0.325
Hypertension (%)	68 (42.0)	26 (43.3)	42 (41.2)	0.726
Diabetes mellitus (%)	49 (30.2)	19 (31.7)	30 (29.4)	0.448
Chronic heart disease (%)	43 (26.5)	22 (36.7)	21 (20.6)	0.029
Chronic lung disease (%)	25 (15.4)	6 (10.0)	19 (18.6)	0.175
Cerebral disease (%)	43 (26.5)	17 (28.3)	26 (25.5)	0.715
CKD (%)	17 (10.5)	12 (20.0)	5 (4.9)	0.006
In-hospital mortality	37 (22.8)	25 (42.7	12 (11.8)	0.000
*Clinical presentation*				
qSOFA score	2 (1.2)	2 (2.2)	1 (1.2)	0.000
Heart rate (bpm)	109 ± 26	114 ± 30	106 ± 23	0.061
Systolic pressure (mmHg)	122 ± 27	117 ± 28	124 ± 26	0.112
Diastolic pressure (mmHg)	67 ± 16	66 ± 17	68 ± 16	0.499
Tmax (℃)	39.0 ± 0.9	39.0 ± 1.1	39.0 ± 0.8	0.690
SpO_2_ (%)	95 (88.99)	95 (87.99)	95 (89.99)	0.944
Lac (mmol/L)	1.9 (1.1, 3.8)	2.8 (1.5, 6.0)	1.6 (1.1, 2.7)	0.000
PaO_2_/FiO_2_ (mmHg)	230 ± 94	217 ± 105	238 ± 86	0.211
*Laboratory tests*				
CRP	147.4 (82.3, 200)	200 (126.2, 200)	120 (68.5, 200)	0.000
Hemoglobin (g/L)	103 ± 29	96 ± 32	108 ± 26	0.016
WBC (× 10^9^/L)	12.9 (9.5, 17.1)	15.8 (11.3, 20.2)	11.7 (8.3, 15.4)	0.002
Neutrophils (%)	88.2 (81.7, 92.2)	90.2 (84.5, 93.6)	86.6 (80.1, 90.8)	0.009
PLT (× 10^9^/L)	128 (72, 222)	91 (36, 164)	152 (85, 241)	0.000
PCT (ng/mL)	2.635 (0.422, 8.338)	6.960 (1.870, 25.600)	1.220 (0.204, 4.350)	0.000
SCr at admission (μmol/L)	73.0 (58.5, 120.0)	144.0 (78.5, 231.0)	68.0 (52.5, 91.0)	0.000
SCr Max (μmol/L)	90.0 (64.5, 191.1)	221.0 (149.5, 316.3)	72.0 (56.5, 91.5)	0.000
eGFR (mL/min*1.73m^2^)	67.24 (26.58, 86.25)	21.92 (13.47, 38.54)	84.79 (68.74, 97.19)	0.000
BUN (mmol/L)	10.76 (6.41, 21.61)	24.42 (18.84, 34.48)	7.33 (5.36, 10.66)	0.000
TNI (ng/mL)	0.050 (0.050, 0.052)	0.05 (0.05, 0.160)	0.05 (0.05, 0.05)	0.000
BNP (ng/mL)	196 (65, 521)	419 (100, 1180)	128 (60, 329)	0.002
FBG (mmol/L)	7.48 (6.07, 11.55)	8.14 (6.45, 13.71)	7.41 (5.98, 10.48)	0.134
Albumin (g/L)	29.9 ± 5.9	27.4 ± 4.8	31.5 ± 6.0	0.000
TBil (μmol/L)	15.9 (10.4, 30.0)	20.2 (12.7, 33.3)	15.1 (9.2, 22.4)	0.003
Prothrombin activity (%)	66 ± 18	61 ± 20	69 ± 16	0.008
Fibrinogen (mg/dL)	458 (374, 569)	479 (328, 601)	452 (396, 550)	0.721
d-Dimer (ng/mL)	1833 (532, 3394)	3374 (2174, 7307)	830 (410, 2035)	0.000
Hemoculture positive (%)	27 (16.7)	11 (18.3)	16 (15.7)	0.668
Other culture of bacteria positive (%)	37 (22.8)	16 (26.7)	21 (20.6)	0.442
CRE positive (%)	8 (4.9)	4 (6.7)	4 (3.9)	0.474
Virus positive (%)	13 (8.0)	3 (5.0)	10 (9.8)	0.375
Fungal culture positive (%)	14 (8.5)	7 (11.7)	7 (6.9)	0.388
LVEF (%)	65 ± 9	63 ± 12	66 ± 7	0.174
*Therapies*				
Carbapenems (%)	104 (64.2)	50 (83.3)	54 (52.9)	0.000
Penicillins (%)	76 (46.9)	24 (40.0)	52 (51.0)	0.195
Cephalosporin (%)	58 (35.8)	20 (33.3)	38 (37.3)	0.735
Quinolone (%)	111 (68.5)	43 (71.7)	68 (66.7)	0.600
Aminoglycosides (%)	32 (19.8)	15 (25.0)	17 (16.7)	0.223
Macrolide (%)	5 (3.1)	0 (0)	5 (4.9)	0.159
Sulfonamides (%)	5 (3.1)	2 (3.3)	3 (2.9)	0.613
Tetracycline (%)	4 (2.5)	3 (5.0)	1 (1.0)	0.144
Gram-positive coccal antibiotics (%)	36 (22.2)	15 (25.0)	21 (20.6)	0.560
Antiviral therapy (%)	26 (16.1)	10 (16.7)	16 (15.7)	0.518
Fungal antibiotics (%)	20 (12.3)	13 (21.7)	7 (6.9)	0.012
Dopamine (%)	8 (4.9)	6 (10.0)	2 (2.0)	0.053
Noradrenaline (%)	15 (9.3)	12 (20.0)	3 (2.9)	0.000
Metaraminol (%)	18 (11.1)	13 (21.7)	5 (4.9)	0.002
Glucocorticoid therapy (%)	38 (23.5)	14 (23.3)	24 (23.5)	0.568
NSAIDs (%)	8 (4.9)	1 (1.7)	7 (6.9)	0.260
Use of furosemide (mg/days)	0 (0.20)	20 (0.40)	0 (0.20)	0.000
CVC (%)	22 (13.6)	15 (25.0)	7 (6.9)	0.002
In-hospital surgery (%)	2 (1.2)	1 (1.7)	1 (1.0)	0.608
Mechanical ventilation (%)	15 (9.3)	10 (16.7)	5 (4.9)	0.022
CRRT (%)	6 (3.7)	6 (10.0)	0 (0)	0.000
ECMO (%)	2 (1.2)	2 (3.3)	0 (0)	0.136

Data expressed as mean ± standard deviation, *n* (%), or median (interquartile range)

CKD, chronic kidney disease; SCr, serum creatinine; eGFR, estimated glomerular filtration rate; FBG, fa sting blood glucose; CRE, carbapenem-resistant enterobacteria; LVEF, left ventricular ejection fraction; NSAIDs, non-steroidal anti-inflammatory drugs; CVC, central vein catheterization; CRRT, continuous renal replacement therapy; ECMO, extracorporeal membrane oxygenation. Compared with non-AKI group, *P* < 0.05 was considered significantly

### Discrimination performance and accuracy of biomarkers regarding septic AKI

**Fig. 1:**
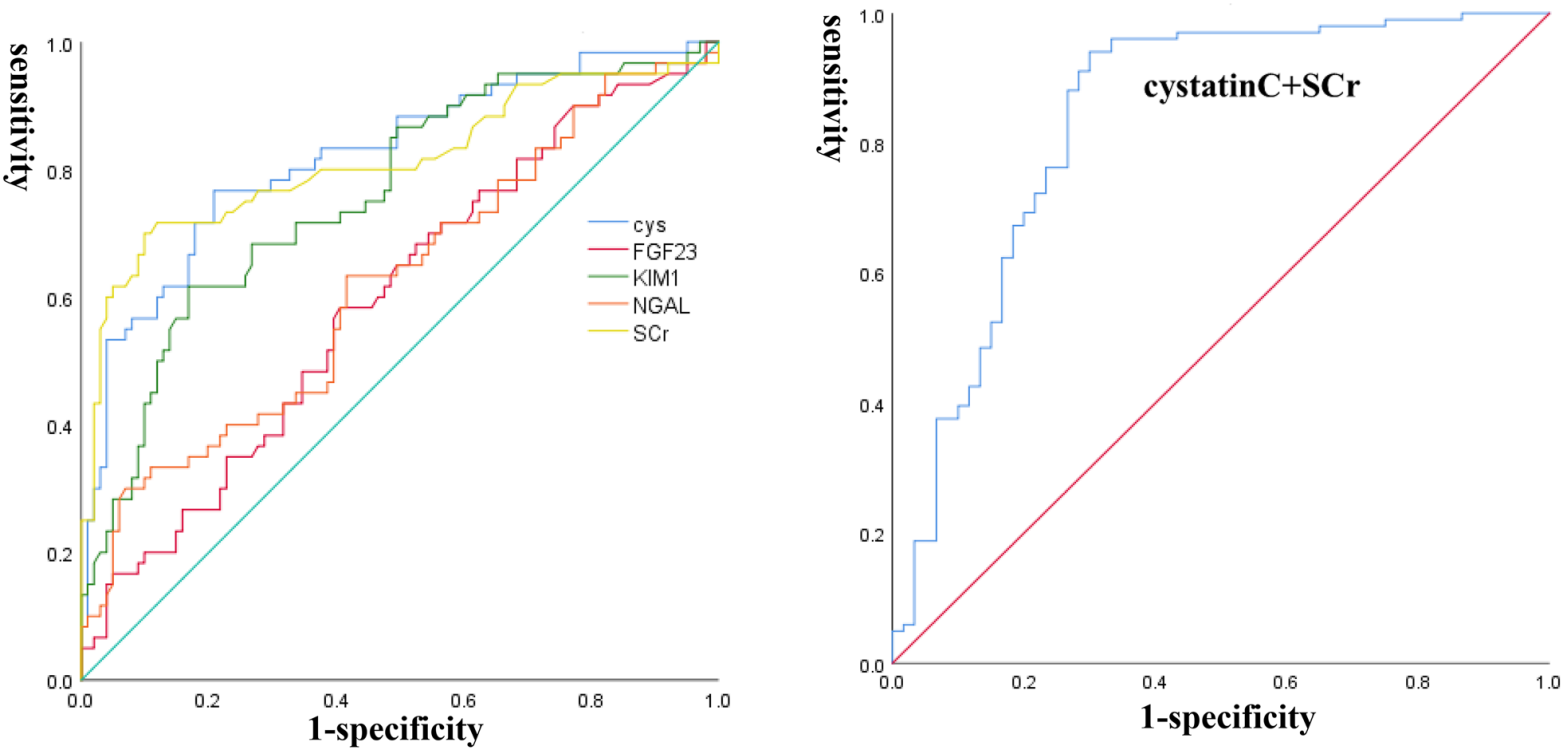
On the left: comparison of discrimination performance of cystatin C, KIM-1, NGAL, FGF23 and SCr regarding the incidence of AKI after sepsis. Cystatin Cis blue, SCr is yellow, KIM-1 is green, NGAL is orange, and FGF-23 is red. The respective AUROC are 0.830 (*P* < 0.001), 0.819 (*P* < 0.001), 0.765 (*P* < 0.001), 0.620 (*P* = 0.021) and 0.596 (*P* = 0.042); On the right: comparison of discrimination performance of cystatin C combined with SCr, which AUROC was 0.847 (*P* < 0.001)

As shown in Table 2, cystatin C, KIM-1, NGAL and FGF23 both increased significantly in AKI group. The ROC curves of biomarkers and SCr on admission are displayed in predicting development of AKI after sepsis in Table 3 and Fig. 1. Areas showed that serum levels of cystatin C had a slightly better discriminative power in prediction of AKI than SCr (AUROC 0.821, 95% confidence interval, 0.752 to 0.891 and AUROC 0.813, 95% confidence interval, 0.736 to 0.890, *P* < 0.001, respectively). In addition, AUROC of cystatin C combined with SCr was up to 0.847 (sensitivity 0.950, specificity 0.700). The cut-off value for cystatin C in predicting AKI was 10.4 μg/ml, sensitivity and specificity values of cystatin C in predicting AKI in sepsis patients were 0.767 and 0.802. The cut-off value of KIM-1 was 135.7 pg/ml and its sensitivity and specificity were 0.617 and 0.832 (AUROC 0.760). NGAL’s cut-off value was 95.6 ng/ml, of which the sensitivity and specificity were 0.300 and 0.931, respectively. The sensitivity and specificity were 0.583 and 0.594 for FGF-23, and its AUROC was 0.596. NGAL, KIM-1 and FGF-23 did not display preferable diagnostic values compared with SCr.

**Table 2:** Early biomarkers in sepsis patients

	Total (*n* = 162)	AKI group (*n* = 60)	Non-AKI group (*n* = 102)	*P* value
Cystatin C (μg/mL)	9.6 (7.4, 15.1)	16.48 (10.45, 22.68)	7.98 (6.65, 10.19)	0.000
NGAL (ng/mL)	43.97 (20.68, 102.24)	44.0 (20.7, 102.2)	30.8 (16.8, 65.0)	0.011
KIM-1 (pg/mL)	87.2 (46.8, 172.7)	173.7 (69.9, 363.5)	58.2 (32.6, 119.3)	0.000
klotho (pg/mL)	92.2 (45.4, 130.7)	96.1 (47.2, 147.1)	89.1 (42.9, 123.2)	0.363
FGF-23 (pg/mL)	300.0 (101.0, 934.4)	380.0 (145.7, 1376.9)	246.0 (84.7, 746.4)	0.046

Data expressed as median (25–75%). Compared with non-AKI group, *P* < 0.05 was considered significantly

**Table 3:** The areas under the receiver operating characteristic curves for serum levels of biomarkers and SCr in admission to predict AKI after sepsis

	AUROC	95%CI	*P* value	Cut-off value	Sensitivity	Specificity
SCr (μmol/L)	0.819	0.742–0.896	< 0.001	100.0	0.601	0.901
Cystatin C (μg/mL)	0.830	0.760–0.897	< 0.001	10.4	0.767	0.802
NGAL (ng/mL)	0.620	0.529–0.711	0.011	95.6	0.300	0.931
KIM-1 (pg/mL)	0.765	0.683–0.838	< 0.001	135.7	0.617	0.832
FGF-23 (pg/mL)	0.596	0.506–0.686	0.042	322.1	0.583	0.594
SCr + cystatin C	0.847	0.777–0.917	< 0.001		0.950	0.700

AUROC, area under the receiver operating characteristic curves; CI, confidence interval

## Discussion

AKI is a common complication in sepsis patients, associated with a longer length of hospitalization and higher morbidity and mortality rate. In this investigation, the incidence of septic AKI is 37.0%, and nearly half of AKI patients (48.7%) have displayed stage 3. Compared to previous studies, the proportion of stage AKI 3 patients in our cohort are relatively higher, which may be related to the population distribution of emergency patients [16]. Septic AKI group tends to be more critical, accompanied with severer inflammation responses, worse cardiac function, and more coagulation dysfunction. Invasive procedures and treatments, use of vasoactive agents and higher grade antibiotic were also observed in the AKI group. On biomarkers associated with septic AKI, we found cystatin C, KIM-1, NGAL and FGF-23 were both elevated in AKI group. We analyzed the precision and discriminative ability of these biomarkers for prediction of septic AKI at admission. Compared with SCr, cystatin C may be a more sensitive biomarker for septic AKI, while other biomarkers did not display diagnostic superiority. Cystatin C combined with SCr in the prediction of septic AKI increased the diagnostic sensitivity prominently.

Cystatin C is a 13-kDa cysteine proteinase inhibitor produced by nucleated cells, and freely filtered by the glomerulus, reabsorbed but not secreted by the renal tubules [6]. Based on the above physiological characteristics, as a renal functional biomarker, cystatin C has been found to be superior to SCr in detecting minor reductions of GFR in a number of studies [17–19]. Newer evidence points towards higher serum cystatin C levels associated with age, gender, lean body mass, fat mass and inflammatory markers like CRP [20]. And some studies reported that severe systemic inflammation caused by sepsis could lead to the elevation of cystatin C, so the prediction of cystatin C in septic AKI is negative [21]. These points are worth noting in generalization of its use to predict septic AKI. However, its use along with SCr seems promising.

Our study population characteristics have shown elevated CRP and PCT levels in AKI group compared to non-AKI group, which might partly explain elevated cystatin C levels in AKI group. Considering the mentioned limitations of study, it is reassuring that serum cystatin C combined with serum creatinine have a better prediction of AKI compared to either of them alone. We observed cystatin C combined with SCr could improve AUROC to 0.847 and enhance its sensitivity to 0.950 with a slight decrease in specificity to 0.700. Since one of the key points in the prevention and treatment of AKI is early detection, the improvement of diagnostic sensitivity is meritorious.

KIM-1 is a type 1 transmembrane glycoprotein containing extracellular mucin and immunoglobulin domains. It is observed that KIM-1 is mainly expressed in proximal tubule cells both in rodents and humans [22]. Basal expression of KIM-1 is low in the normal kidney, but could be dramatically upregulated after ischemia–reperfusion injury AKI and drug-induced AKI models [22, 23]. Moreover, KIM-1 has been reported that it could play a role in renal recovery and tubular regeneration after AKI [24, 25]. However, in clinical diagnostic studies, serum KIM-1 has found only modest results, which is the same as in our study [26]. It is speculated that KIM-1 reflects the injury and repair function of kidney, concentration detection of KIM-1 may not be able to distinguish with high accuracy between injury and recovery of AKI.

NGAL is a 25-kDa protein of the lipocalin superfamily that was originally isolated from human neutrophils, which has been extensively investigated in various AKI phenotypes [27, 28]. Some studies also found that NGAL failed to distinguish patients with an AKI in the setting of sepsis [29]. Although NGAL has been considered as a biomarker of AKI, it is not specific to the kidney and is also produced by other tissues such as neutrophils which synthesized NGAL’s homodimer [27, 28]. Therefore, NGAL may increase systemic infection and inflammation without evidence for AKI. Perhaps due to the physiological characteristics, the performance of NGAL was poor in our study.

Klotho-FGF23 signaling axis was found correlative with AKI [9]. Circulating FGF23 was reported increased and klotho decreased in AKI rodent models, and treatment with klotho could improve kidney damage [9, 13, 30]. However, these two biomarkers were of poor diagnostic value in septic AKI in our study.

There are still some limitations in this study. Firstly, the sample size was relatively small, and all patients were from a single center. Secondly, biomarkers were only detected on admission, although it is more predictable for AKI, but we did not monitor the SCr and biomarkers daily to show continuous changes. Thirdly, the biomarkers need to be proven in a general population without exclusion criteria.

In summary, this study provides a perspective that cystatin C could be a more reliable and sensitive predictor for AKI as compared to SCr. The combination with cystatin C and SCr has an outstanding diagnostic efficiency. Meanwhile, KIM-1, NGAL, klotho and FGF-3 did not display their superiority. Early detection of AKI may allow better therapy and potentially avoid its detrimental clinical outcome. Therefore, an improvement in sensitivity and decent specificity is estimable for emergency physicians in septic AKI’s early diagnosis.

## Data Availability

The datasets used/analyzed in the study are available from the corresponding author on reasonable request.
